# Regulation of *Caenorhabditis elegans *vitellogenesis by DAF-2/IIS through separable transcriptional and posttranscriptional mechanisms

**DOI:** 10.1186/1472-6793-11-11

**Published:** 2011-07-12

**Authors:** Ana S DePina, Wendy B Iser, Sung-Soo Park, Stuart Maudsley, Mark A Wilson, Catherine A Wolkow

**Affiliations:** 1 Laboratory of Neurosciences, NIA Intramural Research Program, NIH, Baltimore, MD 21224, USA

## Abstract

**Background:**

Evolutionary theories of aging propose that longevity evolves as a competition between reproduction and somatic maintenance for a finite pool of resources. Reproduction is thought to shorten lifespan by depleting resources from processes promoting somatic maintenance. Maternal yolk production, vitellogenesis, represents a significant maternal cost for reproduction and is suppressed under genetic and environmental conditions that extend lifespan. However, little is known about the pathways regulating vitellogenesis in response to prolongevity cues.

**Results:**

In order to identify mechanisms that suppress vitellogenesis under prolongevity conditions, we studied factors regulating vitellogenesis in *C. elegans *nematodes. In *C. elegans*, vitellogenesis is depressed in the absence of insulin-like signaling (IIS). We found that the *C. elegans daf-2*/IIS pathway regulates vitellogenesis through two mechanisms. *vit-2 *transcript levels in *daf-2 *mutants were indirectly regulated through a germline-dependent signal, and could be rescued by introduction of *daf-2(+) *sperm. However, yolk protein (YP) levels in *daf-2 *mutants were also regulated by germline-independent posttranscriptional mechanisms.

**Conclusions:**

*C. elegans *vitellogenesis is regulated transcriptionally and posttranscriptionally in response to environmental and reproductive cues. The *daf-2 *pathway suppressed vitellogenesis through transcriptional mechanisms reflecting reproductive phenotypes, as well as distinct posttranscriptional mechanisms. This study reveals that pleiotropic effects of IIS pathway mutations can converge on a common downstream target, vitellogenesis, as a mechanism to modulate longevity.

## Background

According to evolutionary theories of aging, lifespan evolves as a trade-off between the metabolic costs of somatic maintenance with those of reproduction. Reproductive processes such as egg production and progeny rearing are energy-intense and drain resources away from processes that promote somatic maintenance. Studies have provided evidence for a trade-off between reproduction and survival. For example, experimentally increased egg production in wild seabirds is associated with lower rates of postmigratory return to breeding grounds [1,2]. One mechanism by which organisms can regulate the relative burdens of reproduction and somatic maintenance in response to environmental conditions is through phenotypic plasticity of life-history traits, such as growth and reproduction [3]. Plasticity in life-history traits can affect lifespan directly, such as to delay reproduction until environmental conditions improve, or indirectly, as a consequence of elevated stress resistance.

One highly plastic reproductive process is vitellogenesis, the process of maternal yolk production that provides the major nutrient source for developing embryos. Vitellogenin genes are expressed in adult females within tissues specialized for yolk production, such as the insect fat body and avian liver. Vitellogenesis has been well-studied in birds and insects and is hormonally regulated in response to environmental conditions. In insects, vitellogenesis is induced by the coordinate action of 20-hydroxyecdysone and juvenile hormone (JH). Insect JH is regulated, in turn, by insulin-like signaling (IIS). Eggs in *Drosophila *insulin receptor mutants fail to become vitellogenic and this phenotype can be rescued by methoprene, a JH analog [4,5]. In *Aedes aegypti *mosquitoes, vitellogenin gene expression is synergistically enhanced by ecdysone and insulin signaling, via the nutrient sensing protein, TOR [6].

IIS is also required for vitellogenesis in *C. elegans *nematodes [7-9]. In *C. elegans*, IIS is mediated by the insulin receptor-like protein (IR), DAF-2 which transduces signals via AGE-1/PI3K to antagonize the activity of DAF-16, a FoxO transcription factor [10-13]. Under conditions that reduce DAF-2/IR signaling, levels of *vit *gene transcripts and yolk proteins (YP) are both reduced [7,14]. In addition to suppressing vitellogenesis, defective IIS prolongs adult lifespan in *C. elegans *and *Drosophila *[15]. The mechanism by which IIS modulates longevity is likely to be multifactorial and complex [16]. Vitellogenesis appears to be one target of the IIS prolongevity mechanism, possibly for the purpose of reallocating resources to somatic maintenance. Consistently, RNAi knockdown of vitellogenin gene expression could extend lifespan of wildtype adults [7]. Fecundity has been linked to longevity in *daf-2 *mutants. Sixteen *daf-2 *alleles were associated with reduced brood size at nonpermissive temperatures [17]. However, certain alleles, termed class 1, had brood sizes ranging from 85-100% that of wildtype, while class 2 alleles had brood sizes ranging from 60-93% of wildtype. In insects, IIS regulates vitellogenesis hormonally through JH. However, it is not known whether *C. elegans *IIS also regulates vitellogenesis through hormonal effectors or by direct action in the intestine, the site of vitellogenin production.

To address this question, we identified factors modulating *C. elegans *vitellogenesis and then examined whether these factors were modified by IIS or were IIS-independent. The *C. elegans *genome contains six vitellogenin (*vit*) genes. The genes, *vit-1*, *-2*, *-3*, *-4 *and *-5*, all contribute to the pool of YP170, the major yolk protein. *vit-6 *encodes a protein that is processed into YP115 and YP88. Our analyses show that IIS stimulates vitellogenin gene transcription through a sperm-dependent signal, implying the existence of germline-dependent hormonal regulators of vitellogenesis in *C. elegans*. However, yolk protein levels correlated poorly with *vit-2 *mRNA and were regulated by IIS independently of sperm and nutrients. Based on these findings, we propose that *vit *genes may be transcribed in excess and titrated to reflect reproductive needs. Furthermore, IIS deficits appear to suppress vitellogenesis convergently through germline-dependent transcriptional mechanisms and a separate posttranscriptional mechanism.

## Results

### Differential regulation of ***vit-2 ***transcription and translation

**Figure 1:**
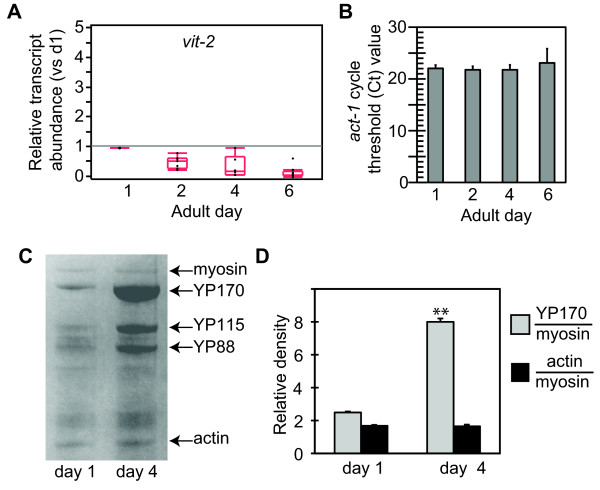
**Regulation of ***vit-2 ***mRNA and yolk protein levels in young adult wildtype ***C***. elegans hermaphrodites**. A) Quantitation of *vit-2 *mRNA in wildtype adult hermaphrodites, relative to *act-1*, normalized to day 1 levels. Levels of *vit-2 *mRNA decline progressively during the first 6 days of adulthood in wildtype hermaphrodites. mRNA levels were measured in independent trials using pools of 30 hermaphrodites at indicated adult ages. Results were normalized to mRNA levels in day 1 adult wildtype hermaphrodites, relative to *act-1*. Results were statistically analyzed and are presented as quantile plots. Black dots indicate results from individual trials. The median value is indicated by a horizontal line within the quantile box plot. The 25th and 75th quantiles are represented by the upper and lower ends of the boxplot, respectively. Whisker lines extending from the box designate the outer-most data point that falls within 1.5 × the range between the 25th and 75th percentiles. Summary statistics are presented in Table 1. B) Absolute values of *act-1 *mRNA as determined using the cycle threshold (Ct). Levels *act-1 *mRNA remained constant between adult days 1-6 in wildtype hermaphrodites (*p *= 0.49, ANOVA). Data are 5 independent measurements at each age. C) Analysis of yolk protein (YP) abundance by SDS-PAGE of total lysates from wildtype hermaphrodites stained with Coomassie blue. Arrows indicate YP170, YP115 and YP88, and myosin and actin which provided standards for YP quantitation. YP levels relative to myosin were elevated in day 4 hermaphrodites compared to day 1, although actin levels remained constant. Gel results are quantified in D). Error bars, standard deviation; ** *p *< 0.01, T-test.

To measure vitellogenesis in *C. elegans *adult hermaphrodites, we examined *vit *mRNA levels and yolk protein (YP) levels. First, quantitative RT-PCR was used to determine levels of the *vit *transcripts contributing to the pool of YP170, the major *C. elegans *yolk protein. We developed primer sets that specifically recognized *vit-2 *or *vit-5*, but not *vit-1*, *-3 *or *-4*, which have very similar sequence. For simplicity, the term "*vit*" may be used to refer to *vit-2/5*. Samples were collected at four time points across the adult reproductive period, between adult days 1 to 6. In wildtype worms, the abundance of *vit-2 *and *-5 *transcripts declined progressively from adult days 1 to 6, resulting in approximately 7-fold reduction over this period (*p *≤ 0.0001, ANOVA) (Figure 1A, Table 1). In comparison, absolute levels of *act-1 *mRNA did not decline over this period, indicating that transcription was not globally reduced and these changes were relatively specific to *vit *genes (*p *= 0.49, ANOVA) (Figure 1B).

**Table 1:** Vitellogenin transcriptional regulation in *daf-2 *pathway mutants

Genotype	Day	vit-2 ^#^	vit-5 ^#^	sod-3 ^#^	Trials
Wildtype	1	1.0	1.0	1.0	17

Wildtype	2	**0.54**(0.20)	0.75 (0.30)	0.90 (0.31)	7 (6^^^)

Wildtype	4	**0.39**(0.35)	0.61 (0.59)	0.77 (0.54)	6 (5^^^)

Wildtype	6	**0.19**(0.16)	**0.23**(0.25)	1.1 (0.96)	13 (12^^^)

*daf-2 (e1368)*	1	1.9 (1.4)	1.5 (1.0)	9.9 (6.9)*	6

*daf-2 (e1368)*	2	0.74 (0.10)	0.55 (1.1)	5.7 (6.2)	3

*daf-2 (e1368)*	4	0.095	0.086	2.53	1

*daf-2 (e1368)*	6	**0.10**(0.08)	**0.10**(0.10)	4.5 (1.2)*	3

*daf-2 (e1370)*	1	1.6 (1.3)	0.86 (0.95)	20.4 (11.5)*	8

*daf-2 (e1370)*	2	0.64 (0.48)	0.16 (0.14)*	21.1 (4.9)*	3

*daf-2 (e1370)*	4	**0.13**(0.14)	**0.04**(0.05)	15.4 (14.5)	3

*daf-2 (e1370)*	6	**0.05**(0.03)*	**0.03**(0.03)*	21.7 (16.1)*	6

*daf-16 (mgDf50);* *daf-2(e1370)*	1	2.1 (1.8)	1.8 (1.8)	1.05 (0.27)	6 (5^^^)

*daf-16 (mgDf50);* *daf-2(e1370)*	2	0.29 (1.0)	0.40 (0.19)	0.56 (0.30)	2

*daf-16 (mgDf50);* *daf-2(e1370)*	4	0.29 (0.43)	0.47 (0.61)	0.24 (0.10)	3

*daf-16 (mgDf50);* *daf-2(e1370)*	6	0.28 (0.29)	0.35 (0.32)	0.34 (0.21)*	2

^# ^mRNA levels relative to day 1 wildtype (SD).

**Bold**, *p *< 0.05 vs day 1, within genotype, T-test.

^^ ^Trials in which *sod-3 *was measured.

^* ^*p *< 0.05 vs wildtype, same day, T-test.

To determine the concordance between *vit *message and protein levels during this period, we also examined yolk proteins directly in wildtype hermaphrodites. Yolk proteins (YP) are the most abundant proteins in the worm and are easily detected in Coomassie-stained crude lysates after polyacrylamide gel electrophoresis [18]. We confirmed the identities of bands corresponding to YP170, YP115, YP88, actin (42 kDa) and myosin (200 kDa) by mass spectrometry. We quantified the levels of each protein by densitometry and calculated abundance relative to myosin, as an internal standard, as well as an independent loading standard run on the same gel. In contrast to the progressive decline observed for *vit-2 *and *-5 *transcripts, YP levels increased in abundance nearly 4-fold by adult day 4 (*p *≤ 0.0001, T-test) (Figure 1C,D). Actin levels did not change over this period. Furthermore, the absolute levels of myosin relative to a loading standard were constant, showing there was no overall change in protein levels. Rather, the increased level of YP170 reflected specific increases in this pool.

**Figure 2:**
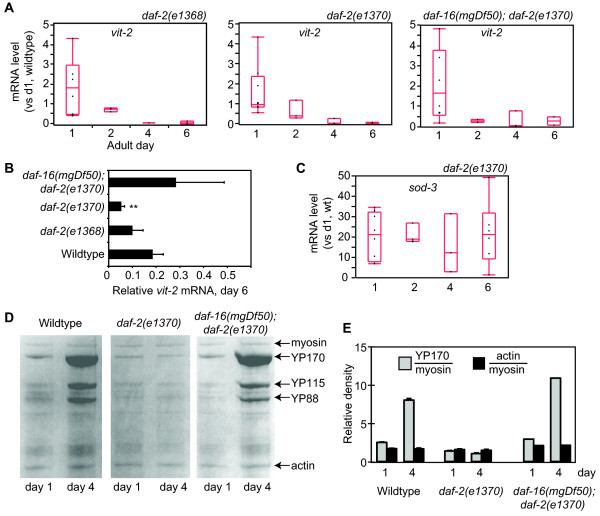
**Suppression of vitellogenesis in ***daf-2(e1370) ***hermaphrodites**. A) *vit-2 *mRNA levels relative to day 1 wildtype hermaphrodites in *daf-2(e1368)*, *daf-2(e1370) *and *daf-16(mgDf50); daf-2(e1370) *adult hermaphrodites. (Left) *vit-2 *mRNA levels were similar to wildtype in *daf-2(e1368)*. (Center) In *daf-2(1370) *hermaphrodites, *vit-2 *mRNA levels were similar to wildtype on days 1-4, but were significantly lower on day 6 (*p *= 0.001, T-test). (Right) The reduction of *vit-2 *mRNA in *daf-2(e1370) *was *daf-16*-dependent and was suppressed in *daf-16(mgDf50); daf-2(e1370) *hermaphrodites. See Table 1 for statistical summary. B) Direct comparison of *vit-2 *mRNA levels in day 6 adults of indicated genotype. Error bars, SEM; ** *p *< 0.001, T-test vs. wildtype day 6. C) *sod-3 *mRNA levels in *daf-2(e1370) *hermaphrodites. There was no statistically significant change between days 1-6 (*p *= 0.78, ANOVA). D) Coomassie-stained SDS-PAGE analysis of total lysates for measuring YP levels. E) Quantification of YP170 and actin abundance relative to myosin, as indicated. In day 1 *daf-2(e1370) *hermaphrodites, YP170 levels were reduced compared to wildtype and *daf-16(mgDf50); daf-2(e1370) *hermaphrodites (*p *< 0.01, T-test). In addition, YP170 increased in day 4 wildtype and *daf-16(mgDf50); daf-2(e1370) *hermaphrodites, but remained low in *daf-2(e1370) *hermaphrodites (*p *< 0.01, *e1370 *vs wildtype, day 4). There were no significant differences in actin levels, relative to myosin, between strains.

We next examined *vit *mRNA levels in *daf-2 *mutant hermaphrodites. Most *daf-2 *mutations prolong adult lifespan but have pleitropic effects on other adult processes. The class 1 *daf-2(e1368) *mutation extends adult lifespan, but does not strongly affect other adult traits, such as fertility, while the class 2 *daf-2(e1370) *mutation is more pleitropic and negatively affects fertility [17]. We observed that *vit-2 *and *-5 *mRNA levels were similar to wildtype in both *daf-2 *mutants on days 1, 2 and 4, but were strongly reduced in *daf-2(e1370) *hermaphrodites on adult day 6 (Figure 2, Table 1). Reduced *vit-2 *transcript abundance in *daf-2 *hermaphrodites was dependent on *daf-16*, which encodes a FOXO transcription factor that is the major target of signaling by the DAF-2/insulin receptor, and *vit *mRNAs were present at normal levels in day 6 *daf-16(mgDf50); daf-2(e1370) *hermaphrodites (Figure 2A,B, Table 1) [12,19,20]. We investigated whether *sod-3*, a direct target of DAF-16, also changed progressively between adult days 1 and 6 [21-23]. Unlike *vit-2*, *sod-3 *mRNA did not undergo a progressive change, but was maintained at high levels in *daf-2(e1370) *mutants across this period (p = 0.92, ANOVA) (Figure 2C, Table 1). This suggests that *vit-2*/*-5 *repression in *daf-2(e1370) *adults occurs through a different mechanism than *sod-3 *activation. In contrast to the progressive decline we observed for *vit *transcripts in *daf-2 *hermaphrodites, YP levels were depressed in *daf-2(e1370) *hermaphrodites for the entire reproductive period (2-fold in day 1 adults vs wildtype, p ≤ 0.001; 8-fold in day 4 adults *vs*. wildtype, p ≤ 0.0001) (Figure 2D,E). YP suppression in *daf-2(e1370) *hermaphrodites was also *daf-16*-dependent. Although YP levels were consistently lower in *daf-2(e1370) *adults, actin was present at normal levels (Figure 2E).

### Sperm stimulate ***vit ***mRNA levels in hermaphrodites

**Figure 3:**
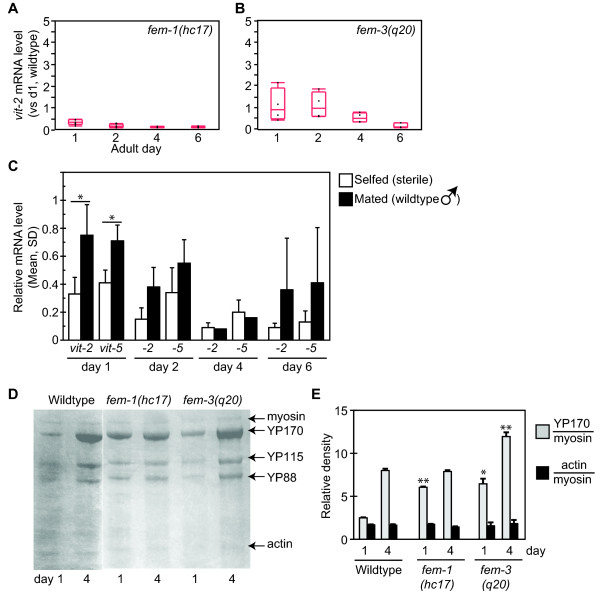
**Regulation of vitellogenesis in sterile mutants**. A, B) *vit-2 *mRNA levels in sperm-deficient *fem-1(hc17) *(A) and oocyte-deficient *fem-3(q20gf) *(B) hermaphrodites, relative to day 1 wildtype hermaphrodites. See Table 2 for statistical summary. C) *vit-2 *and *-5 *mRNA levels in *fem-1(hc17) *hermaphrodites of indicated adult age who were maintained in the absence (unfilled) or presence (filled) of wildtype males (*p < 0.05 mated versus unmated; T-test). D, E) YP levels in sterile *fem-1(hc17) *and *fem-3(q20gf) *adults on days 1 and 4. *p < 0.05, **p < 0.01 versus wildtype hermaphrodites of the same age, T-test.

We next considered whether the progressive decline in *vit *mRNA levels could reflect a physiological change that occurred between adult days 1 and 6. This period encompasses the fertile period, when *C. elegans *hermaphrodites lay a brood of approximately 200 eggs. Sperm abundance limits fertility in wildtype hermaphrodites, and cessation of egg production reflects sperm depletion [24]. To investigate the relationship between reproduction and *vit *expression, we examined *vit *transcripts and YP levels in mutants with reproductive defects. In *fem-1(hc17) *hermaphrodites, which fail to produce sperm [25], *vit-2 *and *vit-5 *mRNA were significantly reduced on adult days 1 and 2 compared to wildtype (Figure 3A, Table 2). These transcripts remained suppressed at days 4 and 6, but were not statistically significantly different than in the wildtype at these ages. In contrast, *vit-2 *and *-5 *mRNA levels were normal in *fem-3(q20gf) *animals, which develop a masculinized germline producing primarily sperm (Figure 3B, Table 2) [26]. The levels of *sod-3 *mRNA were roughly normal in both strains, indicating that the *fem-1 *and *fem-3 *mutations did not have pleitropic effects on *daf-2/daf-16 *pathway activity (Table 2). Thus, *vit *transcriptional suppression may be correlated with deficits in sperm, but not oocyte, production.

**Table 2:** Levels of *vit-2*, *vit-5 *and *sod-3 *mRNA in sterile strains

Genotype	Day	vit-2 ^#^	vit-5 ^#^	sod-3 ^#^
*fem-1(hc17)*	1	0.33 (0.12, 7)*	0.041 (0.09, 7)*	0.93 (0.1, 6)

*fem-1(hc17)*	2	0.15 (0.08, 4)*	0.34 (0.18, 4)*	3.0 (3.7, 2)

*fem-1(hc17)*	4	0.092 (0.03, 3)	0.20 (0.09, 3)	1.2 (1.4, 2)

*fem-1(hc17)*	6	0.094 (0.03, 7)	0.13 (0.08, 7)	0.63 (0.5, 6)

*fem-3(q20)*	1	1.1 (0.76, 4)	1.3 (1.1, 4)	0.98 (1.2, 4)

*fem-3(q20)*	2	1.1 (0.60, 4)	1.1 (0.54, 4)	4.4 (6.8, 4)

*fem-3(q20)*	4	0.53 (0.22, 4)	0.43 (0.18, 4)	1.69 (1.2, 4)

*fem-3(q20)*	6	0.16 (0.12, 3)	0.05 (0.04, 3)*	2.72 (2.31, 3)

*fem-1(hc17) *× wildtype males	1	0.75 (0.22, 4)^^^	0.71 (0.23, 4)	0.53 (0.10, 4)

*fem-1(hc17) *× wildtype males	2	0.38 (0.14, 2)	0.55 (0.17, 2)	0.12 (0.15, 2)

*fem-1(hc17) *× wildtype males	4	0.082 (1 trial)	0.16 (1 trial)	0.24 (1 trial)

*fem-1(hc17) *× wildtype males	6	0.357 (0.37, 3)	0.41 (0.4, 3)	0.20 (0.12, 3)

^# ^Average mRNA level (SD, trials)

* *p *< 0.05 vs. wildtype, same day, T-test.

^^ ^*p *< 0.05, vs. *fem-1(hc17) *unmated, T-test.

To test this hypothesis, we examined whether wildtype sperm could stimulate *vit-2 *mRNA levels in sperm-deficient *fem-1(hc17) *hermaphrodites by measuring mRNA after allowing them to mate with wildtype males. We confirmed that mating rescued *fem-1(hc17) *sterility by the appearance of fertile heterozygous progeny (not shown). Sterile *fem-1 *hermaphrodites co-cultured with wildtype males exhibited a broad range of *vit-2 *mRNA levels which, on average, were elevated compared to unmated *fem-1 *hermaphrodites (Figure 3C, Table 2). On adult day 1, we observed a statistically significant elevation in *vit-2 *mRNA levels from mating (*p *= 0.02; T-test). Mating had no effect on *sod-3 *mRNA (*p *= 0.81). The progressive decline in *vit-2 *mRNA between adult days 1 and 6 was also abrogated by coculture with wildtype males (*vit-2 *mRNA days 1-6: *p *< 0.001 selfed, *p *= 0.17 mated; ANOVA). These results demonstrate that *vit *transcriptional suppression in sterile *fem-1(hc17) *hermaphrodites could be rescued by the introduction of wildtype sperm from mating.

To assess the impact of reduced *vit *transcripts on overall yolk protein production, we examined YP170 levels in sterile *fem-1(hc17) *adult hermaphrodites. In comparison to wildtype adults, YP levels relative to myosin were normal or elevated in *fem-1(hc17) *adults (Figure 3D,E). Yolk protein levels were also elevated in germline-masculinized *fem-3(q20gf) *hermaphrodites. This indicates that the sperm-dependent stimulation in *vit-2/5 *mRNA levels is likely to be dispensable for YP production under normal conditions.

### Deficits in IIS suppress vitellogenesis through two separable effects

**Figure 4:**
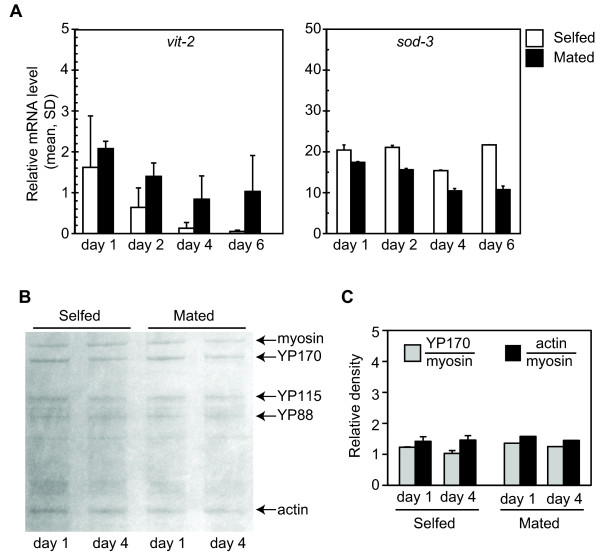
**Vitellogenesis in ***daf-2(e1370) ***hermaphrodites mated with wildtype males**. A) *vit-2 *and *sod-3 *mRNA levels, relative to wildtype day 1, in *daf-2(e1370) *hermaphrodites maintained at 25°C in the absence (unfilled bars) or presence (filled bars) of wildtype males. The levels of *vit-2 *mRNA declined between days 1-6 of adulthood in unmated (self-fertile) hermaphrodites (*p *= 0.016, ANOVA). This decline was abrogated in *daf-2(e1370) *hermaphrodites cocultured with wildtype males (*p *= 0.1, ANOVA). No significant change was detected in *sod-3 *mRNA levels in unmated or mated hermaphrodites (unmated, *p *= 0.17; mated, *p *= 0.88; ANOVA). Similar results were obtained for *vit-5 *mRNA. Unmated, 8 replicates were analyzed for day 1, 3 replicates for days 2 and 4, and 6 replicates for day 6; mated, 3 replicates were analyzed for each age. B) YP levels visualized by Coomassie after SDS-PAGE in *daf-2(e1370) *hermaphrodites maintained as for (A). C) Densitometric quantitation of YP170 and actin (control), relative to myosin, in (B). Although mating with wildtype males could stabilize *vit-2 *mRNA, there was no stimulation of YP levels.

Our analysis showed that *vit-2/-5 *transcriptional suppression in *daf-2(e1370) *hermaphrodites followed the same temporal pattern as in wildtype hermaphrodites. Since *vit-2 *mRNA was stimulated by sperm in *fem-1(hc17) *hermaphrodites, we tested whether wildtype sperm could also enhance *vit-2 *mRNA levels in *daf-2(e1370) *hermaphrodites. When *daf-2(e1370) *hermaphrodites were cocultured with wildtype males, *vit-2 *mRNA levels were stabilized during days 1-6, compared to self-fertilized hermaphrodites (*p *= 0.016 in day 1-6 unmated hermaphrodites; *p *= 0.1 in day 1-6 mated hermaphrodites; ANOVA)(Figure 4A). Similar results were obtained for *vit-5 *mRNA (not shown). Mating had no significant effect on *sod-3 *message levels in *daf-2(e1370) *hermaphrodites, consistent with regulation of these targets through distinct mechanisms (*p *= 0.17 in day 1-6 unmated hermaphrodites; *p *= 0.88 in day 1-6 mated hermaphrodites; ANOVA). As noted for *fem-1*, we detected a broad range of *vit *transcript levels in mated *daf-2(e1370) *hermaphrodites, such that relative differences in *vit *transcripts of mated compared to selfed *daf-2(e1370) *hermaphrodites were not statistically significant at any single timepoint. This variation is likely to reflect differences in mating efficiency between trials [24]. We confirmed that mating occurred by the presence of heterozygous male progeny. Mating did not significantly alter progeny production nor have adverse effects for the mated hermaphrodites, such as death from internal hatching of embryos (not shown). Although mating stimulated levels of *vit-2 *transcripts in *daf-2(e1370) *hermaphrodites, there was no effect on YP (Figure 4B,C). In both mated and unmated *daf-2(e1370) *hermaphrodites, YP170 levels were equivalently reduced in comparison to wildtype.

### Cis-acting requirements for vitellogenin repression in ***daf-2(e1370) ***adults

**Figure 5:**
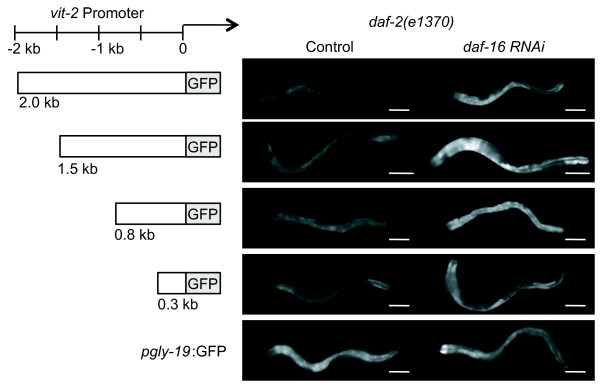
**Expression of ***pvit-2:gfp ***transcriptional reporters in ***daf-2(e1370) ***hermaphrodites in the presence or absence of ***daf-16 ***activity**. Left, schematic illustration of *vit-2 *promoter fragments used to express *gfp*. As a control, GFP expression was examined from the *pgly-19:gfp *transcriptional reporter, containing the 1910 bp *gly-19 *promoter [27]. Right, representative images of GFP fluorescence in *daf-2(e1370) *day 1 adult hermaphrodites maintained on control bacteria (L4440) or *daf-16 *dsRNA-expressing bacteria to induce *daf-16 *RNAi. In all experiments, animals were raised at 15°C to the L4-adult molt and then transferred to 25°C, the nonpermissive condition for certain *daf-2(e1370) *phenotypes. GFP expression from all *vit-2 *promoters was increased under *daf-16 *RNAi conditions, indicating that *daf-16 *activity suppressed expression from each *vit-2 *promoter fragment. GFP expression from the *gly-19 *promoter was unaffected by *daf-16 *RNAi. Bars, 100 microns. Images were collected with identical acquisition settings. Statistical summary presented in Table 3.

To further characterize the basis for YP suppression in *daf-2(e1370) *hermaphrodites, we tested whether a GFP reporter expressed from the *vit-2 *promoter could recapitulate *daf-2 *regulation of endogenous YP. Using an *pvit-2:gfp *reporter containing 2 kb of upstream promoter sequence (*pvit-2(2.0):gfp*), we compared GFP fluorescence in *daf-2(e1370) *day 1 adult hermaphrodites grown on *daf-16 *RNAi or control conditions. The *pvit-2:gfp *reporter was a transgene maintained as a stably-transmitted extrachromosomal array. If the *pvit-2:gfp *reporter were a target for *daf-2 *suppression of vitellogenesis, then we expected *daf-16 *RNAi to block *daf-16 *activity and allow increased GFP expression in a *daf-2(e1370) *background. Consistently, the level of GFP fluorescence was higher in *daf-2(e1370) *hermaphrodites treated with *daf-16 *RNAi than untreated animals (68%, 37%, 24% and 35% increase by *daf-16 *RNAi in 4 independent lines) (Figure 5, Table 3). As a negative control, we found that *daf-16 *RNAi did not affect GFP expression from the intestinal *gly-19 *promoter [27], which is independent of *daf-2*-pathway regulation (-3% and -28% change in *pgly-19*:GFP fluorescence after *daf-16 *RNAi in 2 independent lines).

**Table 3:** Effect of *daf-1 6 *RNAi on *pvit-2:gfp *expression in *daf-2(e1370) *adult hermaphrodites

Reporter	Line 1^	Line 2^	Line 3^	Line 4^
*pvit-2(2.0):gfp*	**68%**(0, 1)	**37%**(0.1, 5)	**30%**(0.2, 5)	**35%**(0, 1)

*pvit-2(1.5):gfp *	**47%**(0.04, 2)			

*pvit-2(0.8):gfp *	26% (0.2, 5)*	**48%**(0.2, 2)		

*pvit-2(0.3):gfp *	**30%**(0, 1)	**40%**(0, 1)	**24%**(0, 1)	

*pgly-19:gfp*	-3% (0, 1)	**-28%**(0, 1)		

^^ ^Relative GFP fluorescence (SD, trials). Data show % change (SD, trials) of average maximum GFP fluorescence from *daf-16 *RNAi versus control in *daf-2(e1370) *hermaphrodites. Positive values indicate increased *pvit-2*:GFP fluorescence associated with reduced *daf-16 *activity from RNAi. Bold indicates GFP intensity was significantly different in *daf-16 *RNAi-treated animals in all trials (*p *< 0.05, T-test).

^* ^GFP fluorescence intensity increased statistically significantly in 3/5 trials (*p *≤ 0.05, T-test). In the remaining 2 trials, % GFP intensity change on *daf-16 *RNA was +5% (*p *= 0.3) and +6% (*p *= 0.6)

Using GFP reporters expressed from subfragments of the 2-kb *vit-2 *promoter, we identified a cis-regulatory region responsive to *daf-16 *activity. Deletions of 0.5, 1.2 and 1.7 kb upstream of the *vit-2 *translational start sequence did not disrupt *daf-16*-dependent repression of *pvit-2*:GFP expression in *daf-2(e1370) *hermaphrodites (Figure 5 Table 3). Thus, *daf-16 *regulation of YP requires only a short 300-bp *vit-2 *promoter, but not additional upstream sequences. The 300-bp *vit-2 *promoter contains binding sites for three transcription factors that direct vitellogenenin expression in the adult hermaphrodite intestine: the ELT-2 GATA factor, the male-specific transcriptional repressor MAB-3, and an unidentified factor which binds to VPE2 repeat sequences in the *vit-2 *promoter [28-30]. DAF-16 recognizes its transcriptional targets through a binding site with consensus GTAAAc/tA [22]. Although the *vit-2 *promoter contains this sequence within the 5-kb upstream of the translational start [7], we did not detect any matches within the 300-bp promoter fragment sufficient for *daf-16*-mediated repression.

We also examined *gfp *mRNA levels in animals containing an integrated *pvit-2(2.0):gfp *transgene. As for the endogenous *vit-2 *locus, *gfp *mRNA was not detectable in wildtype or *daf-2(e1370) *adults immediately after the L4-adult molt (day 0), but increased dramatically by adult day 1 (Table 4). Levels of *gfp *mRNA were similar in day 1 wildtype and *daf-2(e1370) *adults (*p *= 0.771, T-test, 3 trials). This observation suggests that, like endogenous *vit-2*, the *pvit-2:gfp *transgene was not subject to transcriptional suppression at this time point. This is consistent with the conclusion that *daf-16 *activity in *daf-2(e1370) *adults acts to repress GFP levels at a posttranscriptional step.

**Table 4:** Levels of *pvit-2:gfp *mRNA in wildtype and *daf-2(e1370) *adults

	Wildtype		daf-2(e1370)	
**Adult day**	**vit-2^^^**	**gfp^^^**	**vit-2^^^**	**gfp^^^**

0	0.007	0.001	0.008	0.001

1	1.00 (0)	1.00 (0)	2.60 (0.566)^#^	1.10 (0.503)^∞^

Day 0, 1 trial; day 1, 3 trials.

^ Mean mRNA level (SD); mRNA level relative to *act-1*, normalized to wildtype day 1 hermaphrodites.

# *p *= 0.04, vs. *vit-2 *mRNA in wildtype day 1, T-test.

^∞ ^*p *= 0.8, vs *pvit-2:gfp *mRNA in wildtype day 1, T-test.

## Discussion

This study's goal was to characterize the effect of *daf-2 *pathway activity on vitellogenesis in *C. elegans*. Our findings corroborate previous reports showing that *daf-16 *activity suppresses vitellogenesis at both the transcriptional and translational levels [7-9,14]. However, our temporal analysis of this suppression do not support a simple model where DAF-16 acts as a direct transcriptional repressor of *vit *genes. First, YP levels were reduced in a *daf-16*-dependent manner in young adults, although *vit *mRNA levels were normal or elevated at this stage. Second, wildtype sperm stimulated *vit-2 *mRNA non-autonomously in *daf-2 *hermaphrodites, but failed to increase YP levels. Third, we observed *daf-16*-dependent suppression of *pvit-2(0.3 kb):gfp *expression, although this promoter fragment lacks DBE-like DAF-16 binding sites, with the caveat that DAF-16 might bind to non-DBE sites in the *vit-2 *promoter [31]. Finally, we note that *vit *genes were not represented among 103 direct DAF-16 targets identified by chromatin immunoprecipitation [23].

**Figure 6:**
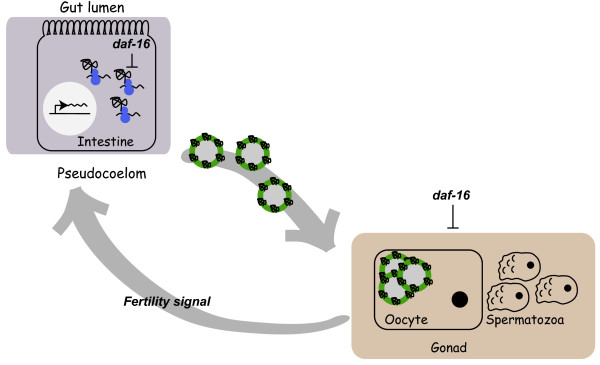
**Model of factors regulating ***C***. elegans vitellogenesis**. In intestinal cells of adult hermaphrodites (left), *vit *genes are transcribed and translated into yolk proteins (YP), which function as ApoB-100-like lipid transport proteins for mobilization of nutrients from the intestine to the oocytes. After synthesis in the intestine, YP are exported into the pseudocoelomic space and taken up by oocytes in the gonad (right). *daf-16 *activity appears to affect vitellogenesis through two separable pathways. In intestinal cells, *daf-16 *activity represses YP production at a posttranscriptional level. In addition, increased *daf-16 *activity in *daf-2(e1370) *hermaphrodites suppresses a germline-dependent signal that stimulates *vit-2 *mRNA production. The *daf-16*-dependent regulation of germline-dependent *vit-2 *mRNA stimulation may reflect *daf-2 *and *daf-16 *regulation of germline proliferation [22].

Since these data do not seemingly support the simple model that DAF-16 functions as a transcriptional repressor at the *vit *loci, we propose an alternative model whereby *daf-16 *represses vitellogenesis through two parallel pathways that independently regulate *vit *transcription and translation (Figure 6). At the transcriptional level, increased *daf-16 *activity in *daf-2(e1370) *adults was associated with a progressive decline of *vit *mRNA which mirrored a similar progressive decline of *vit *mRNA in wildtype adults. We also found that *vit *mRNA levels were low in a feminized mutant unable to produce sperm, but were normal in a masculinized mutant that produces sperm but not oocytes. Together, these observations suggest a role for sperm in stimulating *vit *mRNA levels in early adulthood. The *daf-2 *pathway promotes germcell proliferation and *daf-2(e1370) *hermaphrodites have low fertility at 25°C [17,32,33]. Because the introduction of wildtype sperm could stimulate *vit *mRNA levels in *daf-2(e1370) *hermaphrodites, we theorize that *daf-2(e1370) *hermaphrodites are deficient in a sperm-derived signal that simulates *vit-2/-5 *gene transcription.

In contrast to the progressive decline of *vit *mRNA in *daf-2(e1370) *hermaphrodites, yolk protein production was suppressed constitutively throughout adulthood. This observation suggests a shift of translation regulation. Consistently, GFP expression from the *pvit-2(2.0):gfp *transcriptional reporter was suppressed in a *daf-16*-dependent manner, although *gfp *mRNA levels were comparable in wildtype and *daf-2(e1370) *hermaphrodites. When under stress, cells respond by upregulating translation of stress-resistance proteins, such as heat-shock proteins and chaperones. At the same time, cells repress translation of non-essential proteins to conserve energy supplies [34]. One interpretation of our results is that *daf-2(e1370) *adults undergo a similar translational switch to repress YP synthesis in early adulthood. Genetic results indicate that such a shift in translational regulation should be regulated by one or more DAF-16 target genes. It is not currently known if specific DAF-16 targets may regulate translation. However, MS analysis of 1685 proteins in *daf-2(e1370) *and wildtype adults identified 86 proteins with differential abundance, only 35 of which had been previously identified as DAF-16 target genes [14]. While the remaining 51 proteins might be novel DAF-16 targets, as proposed [14], it is also possible that these might represent additional secondary targets of a *daf-16*-dependent shift in translational regulation. We note that global translational reprogramming in response to thermal stress (35°C, 2 hours) occurs normally in *daf-2(e1370) *adults [35]. Thus, *daf-2/daf-16 *pathway activity may regulate translational reprogramming at 25°C, a relatively nonstressful condition, but not under severe stress, such as at 35°C.

We were surprised to observe significant discordance between *vit *transcript and YP levels under the conditions we examined in the course of this study. In wildtype adults, YP170 increased during adulthood although *vit-2 *and *-5 *mRNA decreased. Furthermore, YP170 was reduced in *daf-2(e1370) *day 1 adults which contained normal levels of *vit-2 *and *-5 *mRNA. We acknowledge the caveat that other *vit *loci (*vit-1*, *-3 *and *-4*), which contribute to the YP170 pool, may have increased expression to make up for the decline in *vit-2 *and *-5*. However, other studies have observed these 5 loci to be expressed at similar levels [8] (S.K. Rao & C.A.Wolkow, unpublished results). In addition, alternative regulation of these loci could not account for the discrepancy between *vit *mRNA and YP170 in day 1 *daf-2(e1370) *adults.

Discrepancies between mRNA and protein levels have been observed under other conditions. Studies in yeast have examined the correlation between transcript and protein abundance on a large scale. With the exception of highly expressed genes, these studies report little correlation between relative levels of mRNA and protein, concluding that transcript levels may be inadequate reflections of the true cellular composition [36]. One factor that could contribute to discordance between *vit *transcript and YP levels is the potential accumulation of YP within embryos in the hermaphrodite uterus or the pseudocoelomic body cavity. Thus, older hermaphrodites containing more eggs in the uterus might also contain more yolk in bulk lysates. However, this explanation does not account for our finding that *fem-3(q20) *hermaphrodites, which fail to make oocytes, contained normal levels of YP170. In this strain, YP are likely to accumulate in the pseudocoelom. Rather, we hypothesize that *vit *genes are transcribed at high levels on the first day of adulthood, in anticipation of reproductive needs. Then, as reproduction proceeds over the next 3-5 days, *vit *mRNA levels are titrated downward in response to actual reproductive demand. Providing excess *vit *transcripts in early adulthood may ensure adequate YP synthesis capacity for maximum reproductive rate.

## Conclusions

Vitellogenins have been repeatedly identified as targets repressed by *daf-16 *activity and downregulated under prolongevity conditions [7-9,14]. Furthermore, vitellogenin downregulation by RNAi was itself sufficient to extend adult *C. elegans *lifespan [7]. These findings indicate that longevity and somatic maintenance in *daf-2 *mutants is likely to reflect combinatorial effects of protective enzyme induction and reproductive suppression. Our studies show that *daf-2 *pathway deficits suppress vitellogenesis through two separable mechanisms. This reveals new insight that longevity assurance in *daf-2 *mutants can result from convergent regulation of vitellogenesis by pleitropic phenotypes in these mutants.

## Methods

### Strains and growth conditions

The strains used in this study were N2 Bristol [wildtype], DR1572 [*daf-2(e1368)*], CB1370 [*daf-2(e1370)*], CY312 [*daf-16(mgDf50*); *daf-2(e1370)*], BA17 [*fem-1(hc17)*], JK816 [*fem-3(q20)*], CY625 [*daf-2(e1370); bvIs7 (pvit-2:gfp; gcy-7:gfp)*]. Strains were obtained from *Caenoharbditis *Genetics Center at the University of Minnesota. All strains were maintained at 15°C on nematode growth medium (NGM) agar plates seeded with OP50 *E. coli *strain following standard protocols [37]. For mating assays, animals were cultured from embryos at 15°C for 4-5 days to the young adult (day 0) stage. Mating assays were carried out using at a ratio of 1 hermaphrodite to 2 males and then shifted to 25°C. 30 hermaphrodites were harvested from mating plates on days 1, 2, 4 and 6 for Q-PCR analysis. For nutrient deprivation experiments, young adult animals (day 0) raised at 15°C were shifted to 25°C for 24 hours (day 1). Day 1 animals were kept on NGM plates with OP50 bacteria (fed) or transferred to plates without food for 6 hours (fasted). Worms were then harvested and processed for Q-PCR analysis or protein gel analysis.

### Quantitative real time RT-PCR

Worms were allowed to lay eggs on NGM agar plates and grown at 15°C for 4-5 days to obtain synchronized populations. Young adult (YA) worms were then shifted to 25°C and harvested on day 1 (fertile adult), day 2, day 4 and day 6 (post-reproductive adult) by picking 30 worms into M9 Buffer. Total RNA was isolated using Absolutely RNA Miniprep Kit (Stratagene, La Jolla, CA). cDNA was synthesized using Stratascript First-Strand Synthesis System (Stratagene). Triplicate 25 μl quantitative real time RT-PCR (qPCR) reactions were set-up in 96-well plates using 2 × SYBRGreen master mix (Applied Biosystems, Foster City, CA), and reactions were run on MJ Research Opticon thermal cycler (BioRad, Hercules, CA). Data was analyzed using the Ct method and relative mRNA levels is reported as the mRNA abundance of each gene relative to the mRNA abundance of the control gene, *act-1 *(β-actin). The following qPCR primers were used: *vit-2*, 5'-GACACCGAGCTCATCCGCCCA and 5'-TTCCTTCTCTCCATTGACCT; *vit-*5, 5'-GGCAATTTGTTAAGCCACAA and 5'-CCTCCTTTGGTCCAGAAACCT; *sod-3*, 5'-CCAACCAGCGCTGAAATTCAATGG and 5'-GGAACCGAAGTCGCGCTTAATAGT; *act-1*, 5'-CCAGGAATTGCTGATCGTATGCAGAA and 5'-TGGAGAGGGGAAGCGAGGATAGA; *gfp*, 5' CTGGAGTTGTCCCAATTCTTG and 5'- AAGCATTGAACACCATAACAGAAA. Results of individual trials were statistically analysed using JMP 7.0 (SAS, Cary, NC) to obtain *p*-values of significance using one-way ANOVA and T-test.

### Protein analysis

Worms were allowed to lay eggs on NGM agar plates and grown at 15°C for 4-5 days to obtain synchronized populations. Young adult (YA) worms were then shifted to 25°C and harvested on adult days 1 and 4 by picking 100 worms into M9 Buffer, diluted in 2 × Laemmli Sample Buffer (Bio-Rad) containing beta-mercaptoethanol and incubated in 70°C water bath for 15 min. The worm mixture was vortexed every 5 min during the incubation and spun for 5 min to remove insoluble precipitates prior to loading on a NuPAGE 4-12% Bis-Tris gel (Invitrogen). Gels were stained and destained following standard protocols. Protein bands were quantified using ImageJ (NIH Image).

For protein identification, Coomassie-stained protein bands were excised, micro-dissected and destained with 50% acetonitrile (ACN) with 50 mM ammonium bicarbonate. Proteins were reduced with 10 mM DTT for 30 minutes at room temperature (RT) and alkylated with 10 mg/mL iodoacetamide (Sigma) for 20 min at RT. After alkylation, gel pieces were treated with trypsin (Sequencing grade, Promega) in 50 mM ammonium bicarbonate overnight at 37°C. The resultant peptides were extracted from the gel with 0.1% TFA in water and then 0.1% TFA in 70% ACN for 10 minutes, collected in eppendorf tubes and dried using a vacuum drier (Savant, USA). Peptide samples were desalted with ZipTip (Millipore), dried again, and stored at -80°C for LC-MS/MS analysis.

LC-MS/MS analyses were performed using an LXQ linear ion trap mass spectrometer coupled to a Surveyor LC system (Thermo Fisher Scientific). Peptide samples were loaded onto pre-equilibrated analytical columns using a pressure-loader. The capillary-tip-columns were affixed to the MS ion-source system and reverse-phase chromatographic separations were carried out using the following gradient setting; 100% A (0.1% formic acid in water) for 5 minutes; 0-50% B (0.1% formic acid in ACN) for 60 minutes; 50-70% B for 5 minutes; 70% B for 5 minutes. Pump flow rates were controlled to deliver 200 nL/min to the analytical column, which was achieved by splitting pump flow at a 1:1000 ratio. LXQ settings were as follows: spray voltage, 1.6 kV; 1 microscan for MS scans at maximum inject time 10 ms with mass range 400-1400 m/z, 3 microscans for MS/MS at maximum inject time100 ms with automatic mass range. The LXQ was operated in a data-dependent mode, that is, one MS scan for precursor ions followed by four data-dependent MS/MS scans for precursor ions above a threshold ion count of 500 with normalized collision energy value of 35%.

The MS/MS data were analyzed with Mascot software v2.3.0 (Matrix Sciences). MS/MS peak lists were generated using lcq_dta.exe from the BioworksBrowser v3.3.1 SP1 (Thermo Fisher Scientific) with the following options applied: grouping tolerance, 0; intermediate scans, 0; minimal scans per group, 0; precursor charge state analysis, auto. The generated lists were searched against Sprot C. elegans database with the following criteria: enzyme, trypsin (KR/P); missed cleavage sites, 3; peptide tolerance, 1.0 amu; fragment ions tolerance, 0.5 amu; variable modifications, carbamidomethylation (+57Da) and methionine oxidation (+16Da); decoy database, on. To ensure the false discovery rate (FDR) below 1%, the search results were further filtered with various significant p-values.

### *pvit-2:gfp *reporter analysis

The *pvit-2:gfp *reporter was constructed using a 2-kb fragment upstream of the *vit-2 *presumptive ATG start site, which was PCR amplified with primers containing unique BamH*I *and Kpn*I *restriction sites and ligated to pPD95.75 vector (gift of A. Fire, Stanford University). Animals were transformed by standard microinjection-mediated transformation using *pvit-2:gfp *plasmid DNA and *gcy-7:gfp *coinjection marker at a final concentration of 25 ng/μL and 100 ng/μL, respectively. For *vit-2 *promoter deletion analysis, the 2-kb *vit-2 *promoter was digested and re-ligated to remove 5' regions of the promoter and generate smaller fragments. Animals were photographed under epi-fluorescence using a 10 × objective on a Nikon E800 with a Hamamatsu Orca ER camera using Openlab software (Improvision). Exposure times were constant for each trial, although exposure times varied between some trials. For GFP quantification, ImageJ (NIH) was used to determine the maximum pixel intensity for a region-of-interest encompassing the intestine. Individual GFP intensity values were measured for an average of 14 animals/trial (range, 3-23 animals) and the values for *daf-16 *RNAi versus L4440 were compared for each trial of each transgene by T-test (Excel). Percentage changes in GFP fluorescence were calculated from the average maximum GFP intensity measured in each experiment after normalization for exposure time, if necessary.

### RNA-interference by feeding

RNA-interference (RNAi) knockdown of *daf-16 *in *C. elegans *was achieved by feeding animals *E. coli *strain HT115 expressing *daf-16 *dsRNA or contained a control plasmid (L4440) [38]. To obtain synchronous populations, 5-10 adult wildtype or *daf-2(e1370) *adults carrying *pvit-2:gfp *transgenic arrays were allowed to lay eggs for 4-6 hrs on NGM agar plates supplemented with ampicillin (100 μg/mL) and IPTG (1 mM). Animals were exposed to *daf-16 *RNAi from hatching, and grown at 15°C for 4-5 days to YA stage. Animals were then shifted to 25°C and images were captured on days 1, 2, and 4 for GFP fluorescence analysis. GFP fluorescence intensity was measured using ImageJ.

## Authors' contributions

ASD, WBI, MAW and CAW designed the experiments. ASD conducted the experiments, except WBI constructed the *pvit-2:gfp *plasmids and strains and performed GFP expression analysis in Figure 5. SP and SM conducted LC/MS/MS protein identification. ASD and CAW wrote the manuscript

All authors read and approved the manuscript.
